# Chromosome-level genome of the European hamster (*Cricetus cricetus*) and its genome-wide population structure across Western Europe

**DOI:** 10.1186/s12915-025-02384-8

**Published:** 2025-09-15

**Authors:** Tobias Erik Reiners, David Prochotta, Tilman Schell, Carola Greve, Alexander Ben Hamadou, Charlotte Gerheim, Juan-Manuel Ortega, Carsten Nowak, Marcel Nebenführ, Axel Janke

**Affiliations:** 1 Centre for Wildlife Genetics, Senckenberg Research Institute and Natural History Museum Frankfurt, Gelnhausen, Germany; 2 LOEWE-Centre for Translational Biodiversity Genomics (TBG), Senckenberg Nature Research Society, Frankfurt Am Main, Germany; 3 Senckenberg Research Institute and Natural History Museum, Frankfurt, Frankfurt Am Main, Germany; 4 Senckenberg Biodiversity and Climate Research Centre (BiK-F), Frankfurt Am Main, Germany; 5 Institute for Ecology, Evolution and Diversity, Goethe University, Frankfurt Am Main, Germany

**Keywords:** Common hamster, Conservation genomics, Rodent, Conservation breeding, De novo genome, Extinction, Inbreeding, Endangered species, Heterozygosity, Breeding program

## Abstract

**Background:**

The European hamster (*Cricetus cricetus*) was once a pest on European farmland, but its numbers have declined dramatically in recent decades, making it a critically endangered species throughout Europe and beyond. While it is strictly protected by EU law and several conservations, breeding and release programs have been initiated, and little is known about the level of genetic erosion and inbreeding on a European scale.

**Results:**

Here, we present a chromosome-level de novo genome of a female hamster and a first population genomic analysis from the western range of the species’ distribution, using Illumina short reads (10 × coverage) from 34 individuals. The genome is 2.89 Gbp long, with 11 chromosome-level scaffolds and around 600 unplaced scaffolds and scaffolds N50 of 267 Mbp. The genome is above the average length of a mammalian genome and longer than that of other studied hamster species. Four distinct hamster populations with no admixture can be identified, indicating highly isolated populations with limited connectivity. Heterozygosity (Ho) is generally low (< 0.05%, comparable to polar bears) with some exceptions of populations with Ho near zero and a few with Ho as high as 0.2%.

**Conclusions:**

Most dramatically, the genomes of individuals used as founders for conservation breeding programs show exceptionally long runs of homozygosity, questioning its long-term suitability. This study confirms earlier concerns about the dramatically decreasing genetic diversity of the European hamster and provides a basis for future conservation efforts, which require consideration of population genetic factors.

**Supplementary Information:**

The online version contains supplementary material available at 10.1186/s12915-025-02384-8.

## Background

**Fig. 1:**
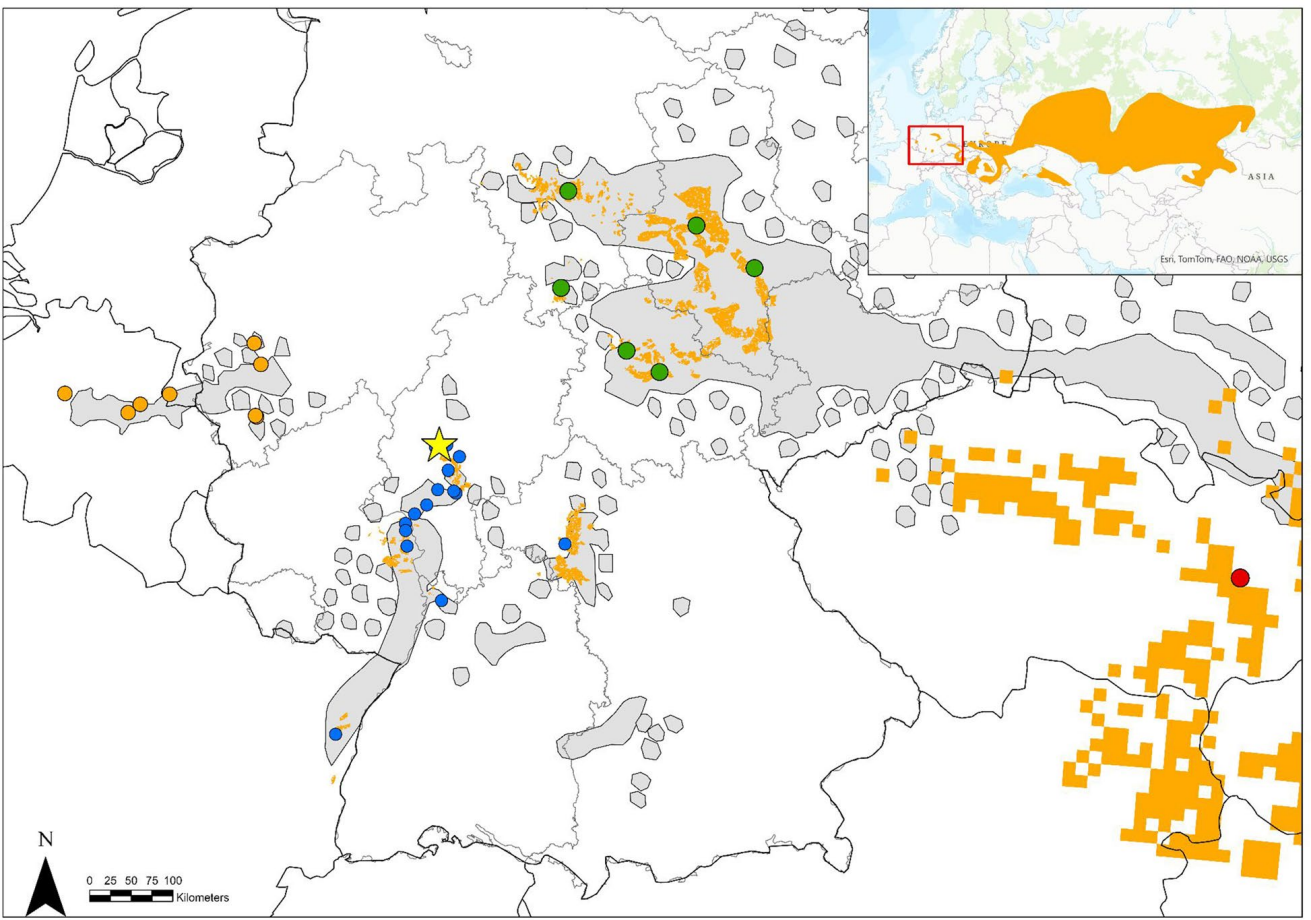
Past (gray) [3] and present (orange) distribution map and sample sites of the European hamster in Belgium and the Netherlands, Germany, France, and the Czech Republic. The red-marked area in the inset map of Europe shows the study areas. In the main map, the orange circles represent samples from the BNN group, the blue circles represent additional samples from the Western lineage, and the green circles represent the samples from the Central lineage. The red circles indicate the locations of three individuals from the Pannonian lineage. The yellow star indicates the sample location of the European hamster individual “Priska,” for which the reference genome was sequenced

The European hamster (*Cricetus cricetus*) has become an iconic species to illustrate the decline of farmland biodiversity in Europe. Once controlled as a pest species with mass outbreaks, forming populations with millions of individuals until the middle of the last century, *C. cricetus* has become one of the most endangered medium-sized rodent species, now listed as “critically endangered” in the IUCN World Red List [1] and only found in increasingly fewer and smaller refugia (Fig. 1). Anyhow, for some countries, a favorable status was reported in the last Article 17 report [2].

The European hamster is the only species of its genus (*Cricetus*) and the largest of all known hamster species (up to 35 cm and 700 g), with a specialized elusive ecology and behavior, including nocturnal and soil-centered lifestyle, performing facultative hibernation [4] and digging of up to 2-m deep burrows. In many regions, it is considered an important prey species [5, 6], as well as a soil engineer contributing to soil fertility, bioturbation, and soil formation [7]. As one of the few remaining wild species of hamsters in Europe, the European hamster is an important part of the agricultural ecosystem and plays a key role in maintaining farmland biodiversity.

Due to pest control in the last century, ongoing agricultural intensification, and urban expansion, the species’ range, reproduction, and population sizes have dramatically declined in the last decades [8]. Although the species has been strictly protected in the European Union in Annex IV of the Habitats Directive since its implementation in 1992, member states have failed to implement proper measures to stop the decline. Since 1992, the species has lost more than 40% of its range in Germany and more than 90% in France, the Netherlands, and Belgium [2]. The increasing habitat loss, the declining and genetically depleted populations, and natural predation have led to several captive breeding and release programs [9–11].

The exact reasons for its dramatic recent population decline are still unknown, but it is assumed that habitat loss and changes in agricultural land use are the main causes, as this species depends on extensive agriculture for food and shelter [8]. Conservation efforts aiming to maintain and restore local hamster populations are focusing on a range of strategies, including habitat restoration, captive breeding programs, reintroduction [10–12], and the development of more sustainable agricultural practices.

Several genetic studies have identified multiple lineages within the western distribution [13–16]. A Western lineage includes the distribution that ranges from Belgium over the Netherlands to North Rhine-Westphalia (further named BNN region), France, and western Germany. Populations in Central and East Germany, spanning the federal states of Lower Saxony, Saxony-Anhalt, Thuringia, and formerly Saxony, are forming the Central lineage [16]. Both the Western and Central lineages are sometimes merged into a Northern lineage [16].

In addition, an allopatric mitochondrial lineage has been discovered in the Czech Republic, Hungary, Romania, Austria, Bulgaria, and Poland [8]. This lineage is believed to have originated from glacial refugia in the Pannonian Basin and will be referred to as “Pannonia.” Despite the population decline observed across the range [8], genetically diverse populations persist in Eastern Europe and Russia [16]. These populations appear to be less affected by the decline, although recent reports indicate a significant decrease in their numbers [8].

In contrast, microsatellite analyses have shown a continuous decline in genetic variation across the western range edge of the species in Western-Central Europe, including France, the Netherlands, and Germany [9, 12, 13, 15, 17, 18]. Specifically, the most north-western population in the BNN region is recognized as genetically depauperate. In 1999, a breeding program started in the Netherlands to preserve the most north-western genotypes [9]. However, the extent of genetic diversity remaining in the founder individuals is only known in microsatellite data and MHC data [19].

To date, the decline of the hamster population and their fragmentation into different lineage clades have been mainly studied based on mitochondrial DNA, microsatellites, and MHC data [13, 15, 17, 19]. While these results provide important information, analyses of whole genome data are required to provide accurate estimates of genetic diversity and inbreeding, which are crucial components when investigating biodiversity loss [20–22]. Furthermore, the knowledge gained from genomic data is crucial in supporting in situ and ex situ population management of the European hamsters, as the number of wild populations declines and the number of conservation breeding programs increases [23].

Here, we have sequenced and de novo assembled a chromosome-level European hamster genome using PacBio HiFi long reads and Hi-C technology. The study is complemented by tenfold Illumina short-read data from a variety of selected western European hamster populations to assess the genome-wide diversity and aid targeted conservation programs.

## Results

The final sequencing and HiFi calling resulted in a total of more than 50 Gb with an N50 of 12,760 bp (Additional file 1: Fig. S1). The total amount of HiFi data corresponds to a theoretical coverage of 17.3 ×, given the genome size estimate of 3.45 Gb [24].

**Fig. 2:**
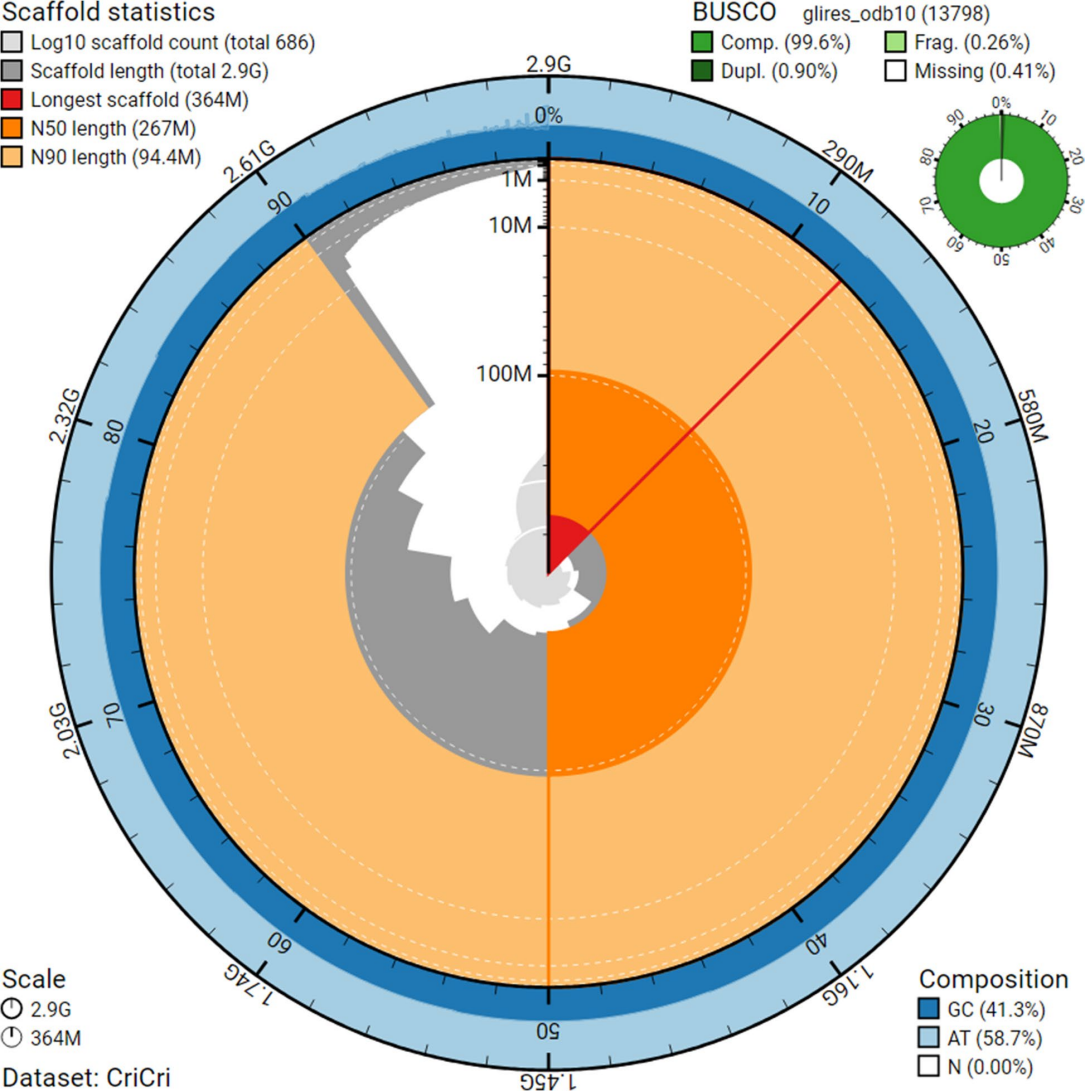
A SnailPlot, summarizing the assembly and Compleasm statistics of the European hamster genome, shows the basic assembly statistics. The radial plot summarizes scaffold statistics (left half) and BUSCO-based completeness assessment (right half) for the assembled genome

The assembly is 2.89 Mbp in length and contains 686 scaffolds with an N50 of 267 Mbp and an L50 of 5 (Additional file 1: Table S1; Fig. S2). The Compleasm analysis (Fig. 2) found 99.6% of BUSCOs from the glires_odb10 database.

**Fig. 3:**
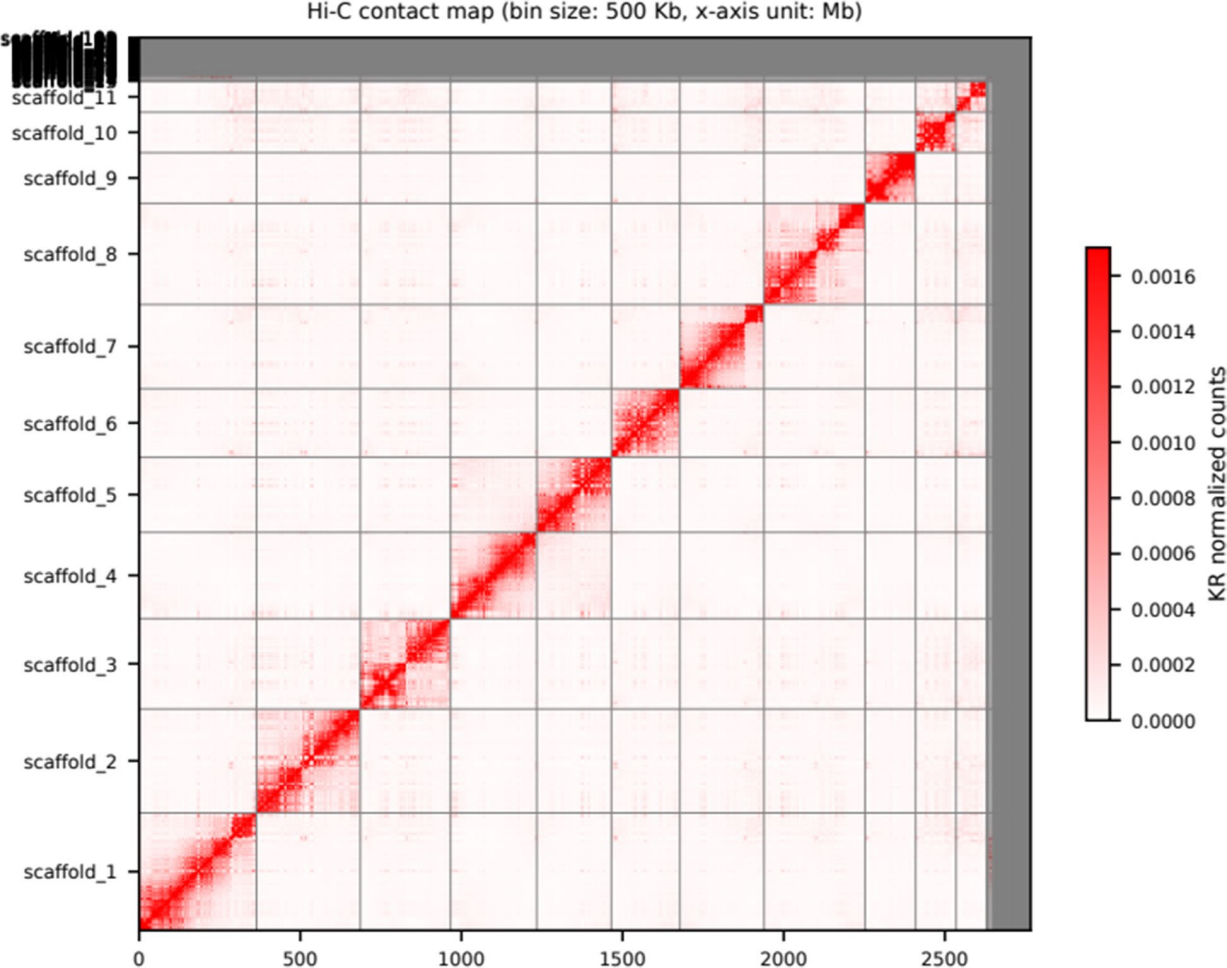
A Hi-C contact density map shows the 11 distinct chromosome-level scaffolds of the European hamster. KR-normalized interaction frequencies are shown for scaffolds 1–11. The strong diagonal signal reflects high intra-scaffold connectivity, consistent with chromosome-scale genome assembly. The map has been manually curated to resolve scaffolding errors. The gray area indicates the unplaced scaffolds

The contact map (Fig. 3) shows the 11 super scaffolds which represent the 11 chromosomes of the European hamster [25, 26] (see also Additional file 1: Table S2).

**Fig. 4:**
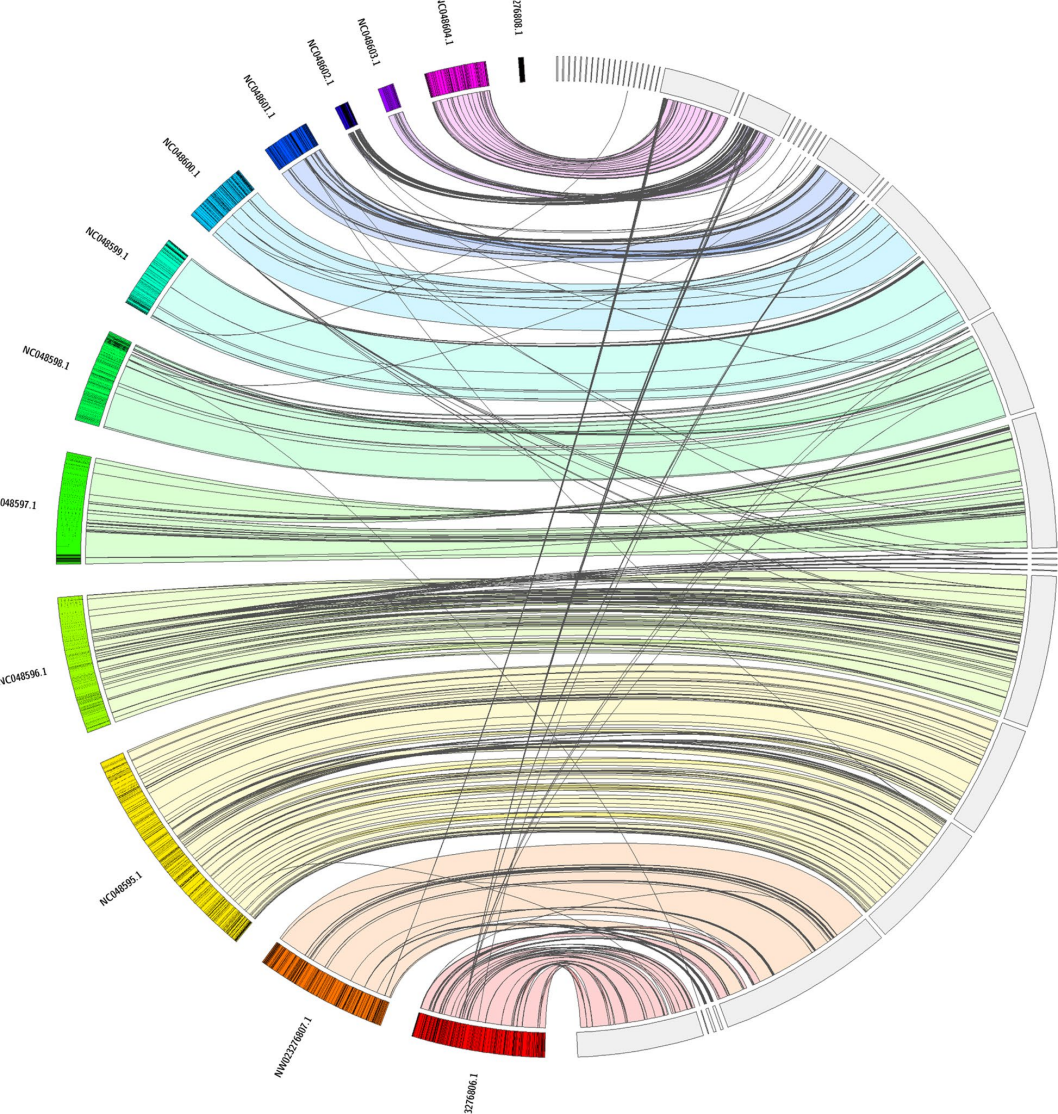
Jupiter plot of the European hamster (left) against the Chinese hamster genome showing relative chromosome synteny despite some (expected) relocations and inversions

The synteny plot (Fig. 4) shows the synteny between the Chinese hamster assembly and our assembly of the European hamster. The chromosomes are mostly syntenic but show that there have been multiple chromosomal splits or joins.

### Population genomic analysis

All individuals could be sequenced with short-read technology and were mapped to the reference genome, resulting in an average mean sequencing depth of 4.5-fold (range of 3.5- to 5.5-fold) after deduplication and filtering (Additional file 1: Table S3).

**Fig. 5:**
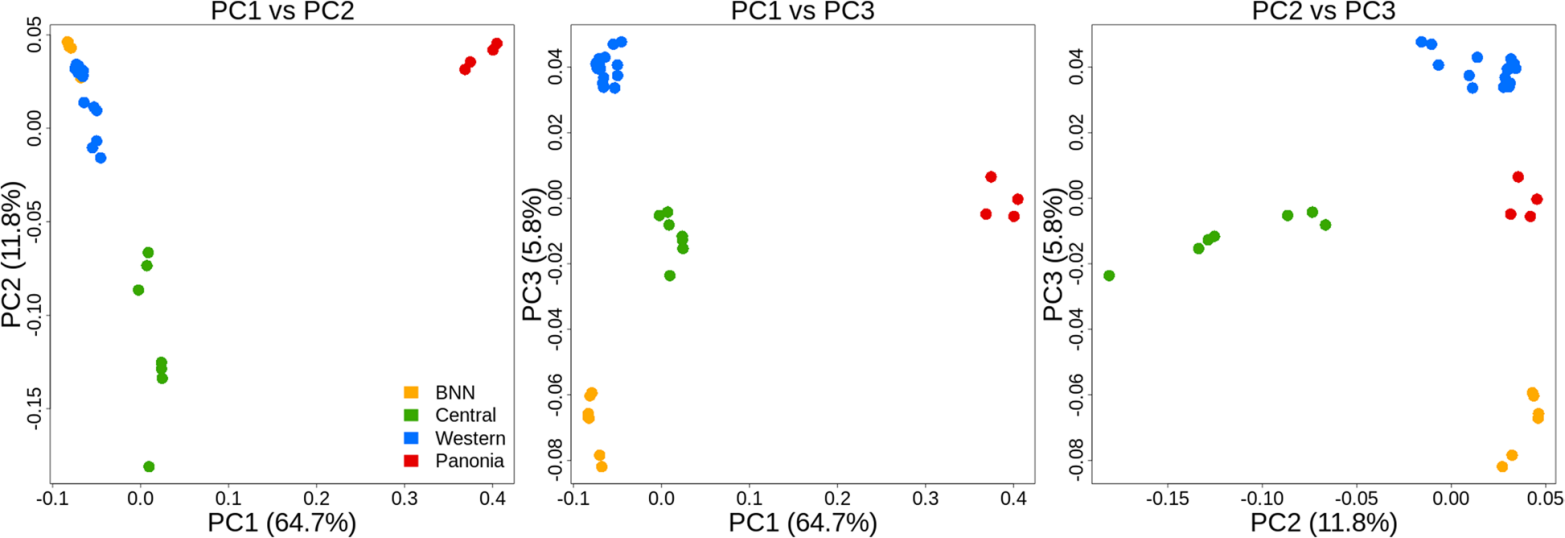
Principal component analysis (PCA) based on 129,944 nuclear SNPs from 35 European hamster individuals. The color scheme for the major populations is the same in the admixture analyses (Fig. 6)

A principal component analysis (Fig. 5) suggested four to seven major clusters, with Czech individuals, as expected, to be the most distant. Individuals from the most westerly BNN region are placed inside the Western lineage but can be clearly distinguished from the other regions when considering the additional PCA axis. Individuals from the Central lineage also form clusters, whereas there is some additional grouping into two to three subclusters.

**Fig. 6:**
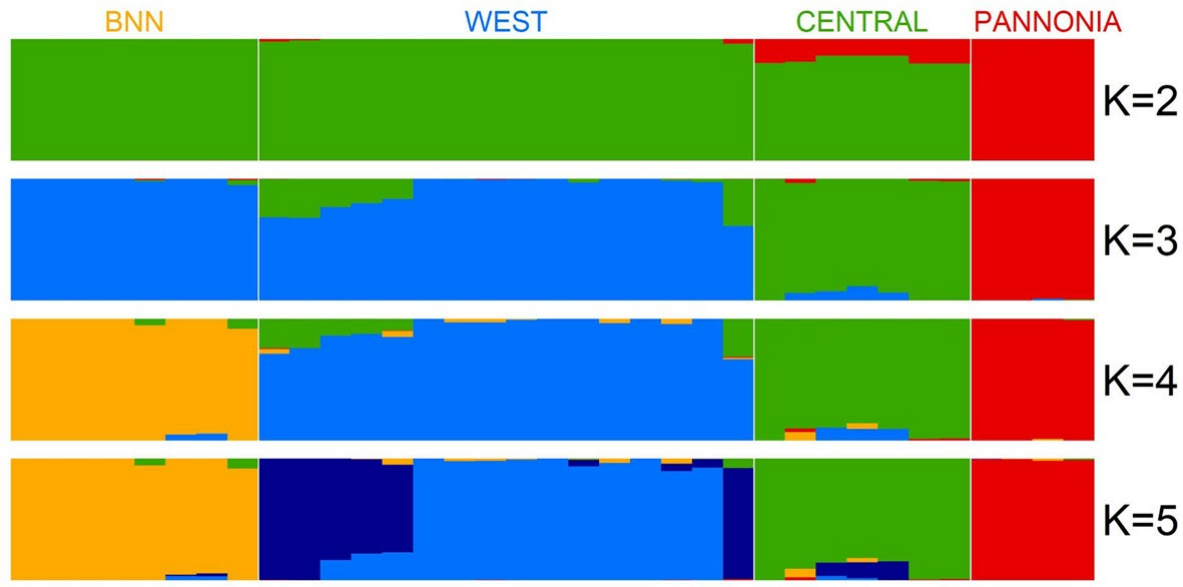
Population admixture results (*K* = 2–5) for 35 European hamster individuals using SamBAr and referencing the sample locations. A cross-entropy analysis finds a minimum of four and five clusters (Additional file 1: Fig. S2)

An admixture analysis separated 35 individuals into 4 groups at *K* = 4 (Fig. 6). The clusters roughly represent the geographic origins of the samples. While the lowest cross-entropy values were obtained at *K* = 4, five clusters (*K* = 5) received a nearly equally low value and separated the individuals from Harxheim, Heer, and Heidelberg (Germany). At *K* = 6, the historical museum samples from 1986 originating from individuals near the city of Worms form their cluster, but admixture and cross-entropy increase, indicating a different genotype at this time (not shown in figure). Another individual originated from the breeding program at Heidelberg Zoo, which is the result of crossbreeding the last wild-caught individuals from Baden-Württemberg with the French hamsters showing a similar pattern.

This grouping is consistent with the PCA analysis, which identifies the same pattern. In addition, the PCA analysis shows a limited variation within the Western population, which is sampled over a wide geographical area. The distinct individuals from the Czech Republic cluster are also very close but come from a small geographical area, indicating that the genomic diversity of individuals from the Czech Republic may be underestimated.

**Fig. 7:**
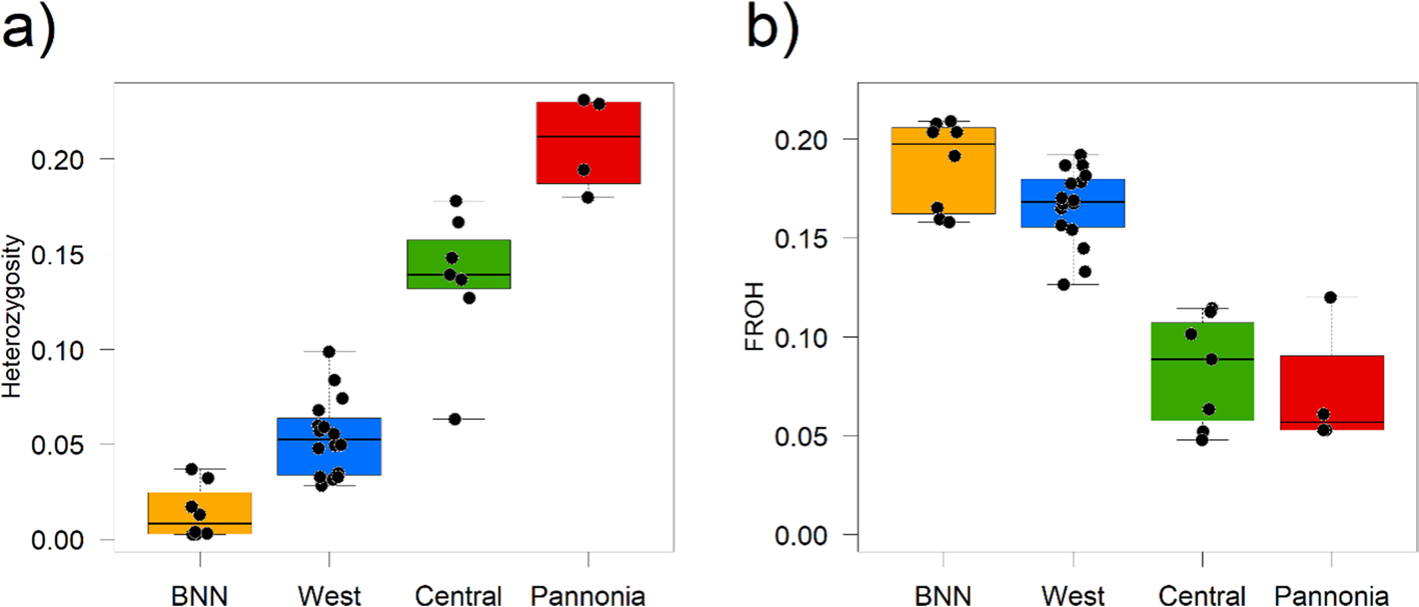
Genomic diversity and inbreeding patterns across populations. **A** Individual heterozygosity (% of heterozygous sites per individual) across four populations: BNN (orange), Western (green), Central (blue), and Pannonia (purple). **B** Inbreeding coefficients based on runs of homozygosity (F-ROH), subdivided by HBD (homozygosity-by-descent) length classes (in generations), with longer ROH indicative of more recent inbreeding. **C** Relationship between the total length of ROH (in Mbp) and the number of ROH segments per individual, color-coded by population. The patterns reveal population-specific differences in both total autozygosity and ROH length distribution, reflecting demographic history and recent inbreeding

The genomes of the 35 hamster individuals show an approximately equal degree of heterozygosity among all individuals, varying from 0.0025% to 0.0231% (Fig. 7A). Analysis of runs of homozygosity (ROH) shows that all hamster populations have a high degree of ROH, indicating general inbreeding, but, except for the Pannonian ones, few long ones, indicating ongoing inbreeding between related individuals (Fig. 7B, C). The BNN population has the lowest degree of heterozygosity, while, in agreement with the low level of F-ROH, the Pannonian individuals still maintain a high degree of genetic variation that is comparable to that of other wild mammal species. The runs of homozygosity (F-ROH) indicate that the Central and Pannonian (Czech) individuals are the least inbred.

## Discussion

The high-quality genome of the European hamster has been assembled to a near chromosome level and has allowed the reconstruction of individual haplotypes to enable genome structure studies such as repetitive elements and chromosomal translocations within the Cricetidae family or even the Muroidea. The reference genome will also be valuable for basic research in medicine and other research fields as the European hamster and closely related genera are used as laboratory animals and research models in infectious disease studies and especially in chronobiology [27, 28].

Chromosome-level data are becoming increasingly available for many wild-life species that attract less attention from research or the public such as the sand eel, okapi, and naked mole rat [29–31]. Combined with population genomics, such data also raise public awareness, as we are only now beginning to understand the basis of biodiversity, delimit new species [32], and detect the effects of anthropogenic impact, such as industrial whaling, on genomic diversity [33]. For instance, understanding the genetic basis of adaptations to specific environments can inform habitat restoration efforts and guide policy decisions to mitigate further biodiversity loss [34, 35]. In addition, genomic data are an important source of information for assessing the extinction risk of many IUCN Red List species, while threats posed by genomic erosion can be addressed through conservation efforts [36].

While genomic studies are providing deep insights into many threatened animal species [37, 38], for some, such as the European hamster, research and conservation have relied only on microsatellite, mitochondrial, and MHC data rather than full genomic studies.

The low heterozygosity of the European hamster, particularly in its western range, is expected from previous studies [12, 15, 19, 39], which have documented a significant decline in genetic variation over the years, likely due to habitat fragmentation, changes in agricultural practices, and reduced population sizes [9, 12]. However, critical information about the historical demography, genome-wide genetic diversity, genetic load, and possible maladaptation is still lacking for the species.

Unfortunately, the steep decline of the species in the wild throughout its range is still ongoing. Due to the obligations of the Habitats Directive and the global IUCN Red List status, the efforts to conserve the species through ex situ captive breeding have increased tremendously over the last two decades with more than 15 captive breeding programs in over 6 countries. Future population management of the species must integrate in situ and ex situ approaches [23]. It is of paramount importance to increase the gene flow between populations, especially for the genetic rescue of small inbred populations [40]. On the other hand, it is equally important that management decisions are based on the best available (genetic) data, currently genomic data. This “genomic” metapopulation management also includes assessing demographic history, the extent of genomic erosion, the risk of outbreeding, and the accumulation of genetic load [38]. If these are not properly addressed, other in situ conservation efforts may be compromised by insufficient genomic diversity.

This study is the first to provide this essential genomic information for sound future management of the European hamster. The first set of genomic analyses of 35 European hamster individuals from the western European range highlighted the strong differences between the lineages, as previously described with mtDNA and microsatellites (see above), but based on genome-wide data. The genomic analysis resulted in a more accurate estimate of genetic diversity, inbreeding, and population divergence.

For the BNN population, the analysis of the last remaining individuals showed that the degree of genetic erosion is extreme (heterozygosity 0.0025, F-ROH: 0.2091). Although single individuals from each of the remnant populations were integrated into the BNN breeding program in the late 1990 s before the population went extinct, it remains uncertain if the founders were sufficient to establish a genetically healthy breeding population already in its 26th generation in captivity. There is an urgent need to re-evaluate the genetic basis of the breeding program using genomic data.

For the Western lineage clade, it is evident that the genomic diversity present in the 1980 s and 2000 s (both highest levels of heterozygosity) is not found in any population today, indicating a strong genomic erosion. Contemporary populations have highly divergent levels of genomic diversity, with some populations showing low inbreeding and high F-ROH, while nearly all populations show signs of severe isolation and inbreeding, with some shifting close to the critical levels of genomic diversity as found in BNN. In one of the impoverished populations, from which the reference individual “Priska” originates, estimates of low genomic diversity led to the implementation of a breeding and release program to reconnect this population with a neighboring population (W06 & W07). This genetic rescue was done in the frame of the project “MetaHamster.” This is the first step towards a sound metapopulation management framework that integrates in situ and ex situ conservation approaches.

For the Central lineage, genomic diversity levels exceed those of the Western population by nearly a factor of 3, highlighting the longer phylogenetic history and larger population sizes, at least in the past. Strong signs of genomic erosion were detected in only one population in the central region (see Fig. 7, population C2). These analyses also led to the complete transfer of this wild population to captivity and genetic rescue with individuals from the neighboring population (C2 & C1). An admixed captive population of > 50 individuals will be used for reintroduction into the former range. A similar approach has been used at Heidelberg Zoo, where the last remaining wild individuals were mixed with French hamsters in the 2000 s (W01 & W02). Genomic monitoring will be required to assess whether the genetic rescue was sufficient.

Attention should be paid to the increasing number of European hamster breeding centers (more than 10 facilities). If the breeding program is based only on a small number of already inbred founders, this could lead to a slow but steady increase of inbreeding, depending on the studbook management, the size of the breeding population, and the origin of founders. Improper genetic management could have irreversible negative effects on genetic health. In particular, constant inbreeding could lead to the fixation of previously heterozygous recessive mutations in emerging runs of homozygosity [33, 41, 42] affecting the long-term fitness of the European hamster.

Comparative analysis of genomic studies in other species provides valuable lessons for the conservation of the European hamster. For example, the Florida panther recovery efforts demonstrate how genetic monitoring and the introduction of individuals from different populations successfully increased genetic diversity, resulting in improved health outcomes [43]. Similarly, the black-footed ferret program used assisted gene flow to increase genetic variability, resulting in a significant population recovery [44]. These successful projects highlight methods that can be adapted for the European hamster.

Finally, the putatively relatively high level of heterozygosity of the Eastern European hamster population makes it a valuable resource for improving the genetic health of hamsters in other regions. A sound genetic management based on the best available genomic data, including historical and contemporary samples, provides a positive prospect. The case of the European hamster may become a role model for how genomics can be translated into conservation practice [45].

## Conclusions

The European hamster (*C. cricetus*), once common, is now critically endangered. This study presents a high-quality reference genome and analyzes genetic data from 34 individuals across the species’ western range. It reveals four genetically isolated populations with very low heterozygosity, indicating severe genetic erosion and inbreeding. Particularly concerning are conservation breeding founders, which show extensive homozygosity. These findings highlight the urgent need to integrate genetic considerations into future conservation strategies.

Chromosome-level reference genomes and population genome sequencing in wildlife will allow a much deeper understanding of biodiversity at the genome level. The sharp decline in the European hamster population over the last decades is not fully understood, and despite conservation efforts, this trend continues. Further studies from a broader range, including Eastern Europe and Russia, as well as detailed studies of local populations and breeding stocks, will be needed, however, as this is only a first and limited attempt to get a first glimpse into the genetic health of the European hamster. Furthermore, the increasing availability of whole-genome analyses will help to avoid targeting the wrong populations for conservation and will help breeding and release programs select the best-suited individuals for the conservation of this iconic European rodent.

## Methods

### Sample origin

To sequence the reference genome, we chose a tissue sample from a female individual “Priska,” who was caught in the wild in 2021 for a captive breeding program in western Germany (Langgöns, Hesse). The hamster “Priska” died during diagnostic isoflurane anesthesia after it was found in bad health condition (weightloss, dyspnea, highly reduced mobility) in the cage. A tissue sample of the animal was preserved in RNA later. The capture and breeding of European hamsters are part of an assisted migration program and approved by the Hessian veterinary authorities on 03/03/2022 and 06/03/2022 (Veterinäramt Gießen §11 TierSchG, Regierungspräsidium Gießen, §8 Abs. 1 TierSchG, §11 TierSchG) as well as the Hessian nature conservation authority on 12/03/2020 (Regierungspräsidium Gießen, document no.: 2020/1061486).

In addition, we resequenced the genomes of 34 additional European hamster specimens from across the species’ western and central European distribution. For this, we collected tissue samples from deceased individuals for standard DNA isolation. Carcasses were found by chance during monitoring and conservation projects. The geographical distance between the sampling sites precludes any kinship between the hamsters sampled. Some historical tissue samples were obtained from museum collections (Additional file 1: Table S3 for sampling locations).

### DNA preparation and sequencing

High molecular weight genomic DNA was extracted from heart tissue according to the protocol of Sambrook and Russell [46]. DNA concentration and DNA fragment length were assessed using the Qubit dsDNA BR Assay kit on the Qubit Fluorometer (Thermo Fisher Scientific) and the Genomic DNA ScreenTape on the Agilent 2200 TapeStation system (Agilent Technologies), respectively. Two SMRTbell libraries were prepared according to the instructions of the SMRTbell Express Prep Kit v2.0 and SMRTbell Express Prep Kit v3.0. The total input DNA was approximately 5 µg per library.

Three SMRT cell sequencing runs were performed in CCS mode using the Sequel System IIe with the Sequel II Binding kit 3.2 (Pacific Biosciences, Menlo Park, CA, USA). The SMRTbell library prepared using the SMRTbell Express Prep Kit v2.0 was loaded twice on the PacBio. Using adaptive loading, two libraries were loaded at an on-plate concentration of 80 pM and one at an on-plate concentration of 90 pM.

To prepare a chromatin conformation capture library, we used the Arima High Coverage Hi-C Kit v01 (Arima Genomics) according to the Animal Tissue User Guide for proximity ligation using approximately 110 mg of muscle tissue from the same individual. The proximally ligated DNA was then converted into an Arima High Coverage Hi-C library according to the Swift Biosciences Accel-NGS 2S Plus DNA Library Kit protocol. The fragment size distribution and concentration of the Arima High Coverage Hi-C library were assessed using the TapeStation 2200 (Agilent Technologies) and the Qubit Fluorometer and Qubit dsDNA HS reagents Assay Kit (Thermo Fisher Scientific, Waltham, MA, USA), respectively. The library was sequenced on the NovaSeq 6000 platform at Novogene (UK) using a 150 paired-end sequencing strategy, resulting in an output of 105 Gb.

For short-read re-sequencing, the same DNA isolation method was applied to different tissues stored at − 20 °C or in > 70% ethanol. Quality checks were performed as described. The genomes of 34 hamster individuals were sequenced by Novogene Europe (Cambridge, UK) on the Illumina NovaSeq 6000 platform (2 × 150 bp, 350 bp insert size) with approximately tenfold coverage.

Total RNA was isolated from the heart, liver, brain, ovary, eye, and gonad from the same individual used to generate the reference genome using TRIzol reagent (Invitrogen) according to the manufacturer’s instructions. The quality and concentration of each extraction were assessed using the TapeStation 2200 (Agilent Technologies) and the Qubit Fluorometer with the RNA BR Reagents Assay Kit (Thermo Fisher Scientific, Waltham, MA, USA). The RNA extractions were then pooled at equal concentrations and sent to Novogene (UK) for Illumina paired-end 150-bp RNA-seq of a cDNA library (insert size: 350 bp) with an expected output of 20 Gb.

### Assembly and scaffolding

HiFi reads were called using a pipeline consisting of PacBio’s tools ccs 6.4.0 (https://github.com/PacificBiosciences/ccs) and actc 0.3.1 (https://github.com/PacificBiosciences/actc) as well as Samtools 1.15 [47] and DeepConsensus 1.2.0 [48]. All commands were executed as recommended in the respective guide for DeepConsensus (https://github.com/google/deepconsensus/blob/r1.2/docs/quick_start.md) except for ccs –min-rq = 0.88, which was set to –all instead. Hifiasm v0.19.8 [49] was used to create the assembly using HiFi and HiC reads with a 45 (-s 45) similarity index. After assembly, we polished the assembly by running one round of Inspector v1.2 [50] utilizing the HiFi reads.

For scaffolding the polished draft genome, we first mapped the Hi-C reads to the 0 using Chromap v0.2.5 [51] and then used YaHs v1.2a.1 [52] with default settings to scaffold the genome. Following this, we created a contact map with Juicer [53] and manually curated the genome with JuiceBox v2.17.00 [54]. The final step was the gap-filling of the genome assembly with TGSGapFiller v. 1.2.1 [55].

### Annotation

The final chromosome-level assembly was checked for repeat content, repeat type, and family composition with RepeatMasker v.4.1.6 [56] and RepeatModeler v.1.0.11 [57]. The genome was first masked with the “rodentia” dataset using the internal Dfam libraries and RepeatMasker RepBase edition.

The masked genome was then analyzed with RepeatModeler. The unknown classifications from the resulting library were classified with repclassifier commit “8b0e5cb” (https://github.com/darencard/GenomeAnnotation/) using RepeatMasker RepBase edition.

For the gene annotation, the masking was run in two steps: first, we used RepeatMasker to hardmask all interspersed elements from the “rodentia” dataset (option: –nolow; convert bases to Ns). Then, we used the de novo repeat library to hardmask all de novo identified interspersed elements.

RNA-seq reads were mapped with STAR [58] to the masked genome assembly to use as additional evidence in GeMoMa [59]. The hard-masked assembly was used as input for GeMoMa, and suitable references were chosen (Additional file 1: Table S4). The GFF file was analyzed with AGAT [60].

### Assembly and annotation quality checks and comparison

The final assembly of *C. cricetus* is compared to the publicly available assemblies of *Cricetulus griseus* (GCF_000223135.1) [61], *Mesocricetus auratus* (GCF_017639785.1) [62], *Phodopus roborovskii* (GCF_943737965.1) [63], and *Phodopus sungorus* (GCA_030556225.1)(Additional file 1: Table S1).

Assembly contiguity statistics were calculated with Quast v5.0.2 [64]. Annotation-related statistics were calculated with a custom script (Additional file 1: Table S5). Completeness regarding single-copy orthologs of the assembly and annotations was assessed with BUSCO v5.5.0 [65] and Compleasm v0.2.5 [66] together with the glires_odb10 (*N* = 13,798) set. The SnailPlot was created with Blobtools and Blobtk [67, 68].

### Reference-based assemblies and variant calling

Illumina short reads of the additionally sequenced specimens were trimmed and filtered using Trimmomatic v0.32 [69]. Subsequently, clean reads were mapped against the final European hamster genome using BWA v0.7.17-r1188 [70] and Samtools v1.15 [25]. Duplicated reads were removed using the Picard MarkDuplicates software (http://broadinstitute.github.io/picard/). All files were filtered for mapping quality and alignment score using Samtools view. Finally, repetitive regions were removed from all files with the help of BEDtools intersect v2.30.0 [71] (Additional file 1: Table S6). The quality of the final files was assessed using Qualimap v.2.2.2-dev [72] (Additional file 1: Table S7).

SNP calling was performed with ANGSD v.0.940 [73] using the default Samtools model for genotype likelihood estimation. Extended BAQ calculation (flag − -baq 2) was enabled, with a minimum mapping and base quality of 30 (− minMapQ 30 − minQ 30). Depth statistics for each individual combined was inferred with Sambamba v.1.0.0 [74]. Optimal maximum and minimum depths were calculated using the site depth distribution of all individuals: median ± the median’s absolute deviation. Sites with a strand bias *p*-value, HWE, and heterozygous bias < 1 × 10^−6^ were removed (− doHWE 1 − hwe_pval 1e-6 − sb_pval 1e-6 − hetbias_pval 1e-6). Only biallelic SNPs, those called with a *p*-value < 1 × 10^−6^ and with less than 10% missingness per individual, were retained (− snps_pval 1e-6 -minInd 32). Genotype likelihood output was enabled to output posterior probabilities of all possible genotypes (− doGeno 8). The analysis results were output in a BEAGLE file and a BCF file (− doGLF 2 − doBcf 1).

### Population genomic analysis

The VCF file was prepared for SambaR v.1.09 [75] R v4.1.3 [76] using plink v.1.9 and vcftools v.0.1.17 [77]. The input was filtered (indmiss = 0.25; snpmiss = 0.1) with adegenet [78, 79].

The data was imported into R and stored in a genlight object using the function “read.PLINK” of the R package adegenet 2.1.5 [78, 79].

The data was filtered using the function “filterdata” of the R package SambaR, with the flags “indmiss = 0.25, snpmiss = 0.1, min_mac = 2, dohefilter = TRUE, and min_spacing = 0.” After filtering, 35 out of 35 individuals were retained, with 129,944 out of 182,140 SNPs retained. This filtered dataset was used for structure analyses, including PCA and admixture.

For the remaining analyses, “snpmiss” was set to 0.1 (no more than 10% SNPs per individual), resulting in 129,971 SNPs.

### Heterozygosity and runs of homozygosity (ROH)

Heterozygosity was calculated by generating a consensus sequence and estimating the folded site frequency spectrum with 100 bootstrap replicates using realSFS embedded in ANGSD [73], using modified versions (“-baq2” instead of “-baq 1”, removal of “-C 50”) of the scripts of Coimbra et al. (2021) [32].

The ANGSD VCF file was used as input for the runs of homozygosity (ROH) analysis. The BCF file was converted to PLINK format using PLINK and then to the Oxford geno format, needed as input for RZooRoH v.0.3.1 [80] (plink − bfile angsd.snps − recode oxford − autosome − out angsd.snps). ROH was inferred separately for each population in RZooRoH v.0.3.1, setting 16 pre-defined classes (15 HBD and 1 non-HBD).

The data was visualized following the scripts by Coimbra et al. (2021) in R for plotting with tidyverse packages [81], viridis v.0.4.2 [82], reshape2 v.1.4.4 [83], RColorBrewer v.1.1–3 [84], and patchwork v.1.1.3 [85].

## Supplementary Information

Additional file 1: Figures S1-S2. FigS1- [HIFISTATS: Length distribution, showing read density vs. read lengths, and statistics at different cutoffs for HiFi reads of the three sequenced SMRT cells]. FigS2 – [Results from a cross-entropy analysis. At K = 4 the entropy reaches a minimum]. Tables S1-S7. TabS1- [Assembly statistics of the de-novo genome of the European hamster individual ‘Priska’]. TabS2- [Chromosome length]. TabS3- [Sample information]. TabS4- [Comparison of de novo hamster genomes and reference species used as a reference by GeMoMa, common name, scientific name, and NCBI accessions]. TabS5- [Annotation statistics]. TabS6- [Results generated from Repeatmasker]. TabS7- [Qualimap/MultiQC - Summary table of mapping and coverage metrics for all sequenced individuals].

## Data Availability

Genome assembly and population genomics samples of *Cricetus cricetus* generated and analysed during the current study are available in the GenBank Number: PRJNA1149967 [86]. The detailed scripts used during the current study are available from the corresponding author on reasonable request. The detailed scripts used during the current study are available from the corresponding author on reasonable request.

## Abbreviations

BCF: Binary variant call format
BNN: Belgium, the Netherlands, and North Rhine Westphalia
DNA: Deoxyribonucleic acid
ddRADseq: Double-digested restriction-site-associated DNA sequencing
EU: European Union
FIS: Inbreeding coefficient
FST: Fixation index
HBD: Homozygosity by descent
HiFi: High fidelity
Hi-C: High-throughput chromosome conformation capture
IUCN: International Union for Conservation of Nature
KR: “Knight-Ruiz” normalization
LD: Linkage disequilibrium
MHC: Major histocompatibility complex
ROH: Runs of homozygosity
SNPs: Single-nucleotide polymorphisms
VCF: Variant call format
