# Supplementary material for: Chromosome-level genome of the European hamster (Cricetus cricetus) and its genome-wide population structure across Western Europe
Source: BMC Biol. 2025 Sep 15;23:276. doi: 10.1186/s12915-025-02384-8 (PMC12439417; doi:10.1186/s12915-025-02384-8)
Supplement: Supplementary file 1 — Additional file 1: Figures S1-S2. FigS1- [HIFISTATS: Length distribution, showing read density vs. read lengths, and statistics at different cutoffs for HiFi reads of the three sequenced SMRT cells]. FigS2 – [Results from a cross-entropy analysis. At K = 4 the entropy reaches a minimum]. Tables S1-S7. TabS1- [Assembly statistics of the de-novo genome of the European hamster individual ‘Priska’]. TabS2- [Chromosome length]. TabS3- [Sample information]. TabS4- [Comparison of de novo hamster genomes and reference species used as a reference by GeMoMa, common name, scientific name, and NCBI accessions]. TabS5- [Annotation statistics]. TabS6- [Results generated from Repeatmasker]. TabS7- [Qualimap/MultiQC - Summary table of mapping and coverage metrics for all sequenced individuals]. [file 12915_2025_2384_MOESM1_ESM.docx]

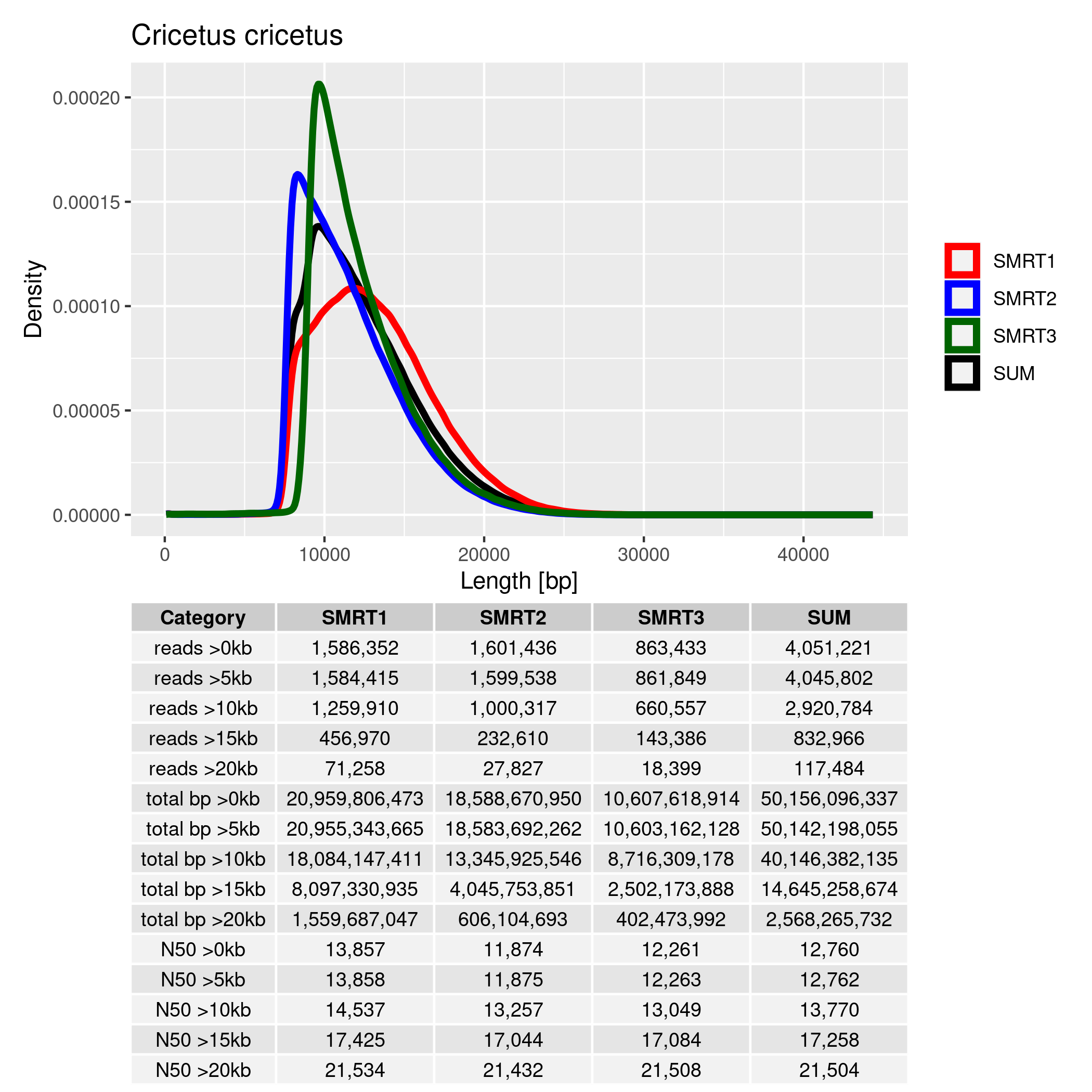


**Fig. S1.** HIFISTATS: Length distribution, showing read density vs. read lengths, and statistics at different cutoffs for HiFi reads of the three sequenced SMRT cells.

**Tab. S1.** Assembly statistics of the de-novo genome of the European hamster individual ‘Priska’

| Assembly | Priska.final | Priska.final_broken |
| --- | --- | --- |
| # contigs (>= 0 bp) | 686 | - |
| # contigs (>= 1000 bp) | 686 | - |
| # contigs (>= 5000 bp) | 678 | 993 |
| # contigs (>= 10000 bp) | 677 | 992 |
| # contigs (>= 25000 bp) | 583 | 895 |
| # contigs (>= 50000 bp) | 528 | 831 |
| Total length (>= 0 bp) | 2898698774 | - |
| Total length (>= 1000 bp) | 2898698774 | - |
| Total length (>= 5000 bp) | 2898685774 | 2898654274 |
| Total length (>= 10000 bp) | 2898680774 | 2898649274 |
| Total length (>= 25000 bp) | 2897001533 | 2896900417 |
| Total length (>= 50000 bp) | 2895167523 | 2894720833 |
| # contigs | 680 | 995 |
| Largest contig | 364397846 | 79516092 |
| Total length | 2898691774 | 2898660274 |
| N50 | 267068797 | 23539883 |
| N75 | 212302528 | 9495126 |
| L50 | 5 | 36 |
| L75 | 8 | 87 |
| # N's per 100 kbp | 1,09 | 0 |

**Tab. S2.** Chromosome length

| Scaffold | Basepairs (bp) |
| --- | --- |
| scaffold_1 | 364397846 |
| scaffold_2 | 321526008 |
| scaffold_3 | 279740146 |
| scaffold_4 | 267068797 |
| scaffold_5 | 231342585 |
| scaffold_6 | 212302528 |
| scaffold_7 | 260916571 |
| scaffold_8 | 313461292 |
| scaffold_9 | 156587254 |
| scaffold_10 | 125071064 |
| scaffold_11 | 94427858 |

**Tab. S3.** Sample information.

| **Label - Collection** | **Label** | **Year** | **Long** | **Lat** | **Coverage** |
| --- | --- | --- | --- | --- | --- |
| W08 | BNN01 | 1999† | 5.7280 | 50.8404 | 3.9 |
| 10/080 | BNN02 | 2009† | 5.4114 | 50.7710 | 3.9 |
| W19 | BNN03 | 2004† | 4.5976 | 50.8452 | 5.2 |
| 09_065 | BNN04 | 2008† | 5.2811 | 50.7138 | 5.2 |
| 06_020 | BNN05 | 2006† | 6.6417 | 51.1865 | 3.9 |
| 2928 | BNN06.1 | 2006† | 6.6661 | 50.6857 | 3.5 |
| H200140 | BNN06.2 | 2014† | 6.6550 | 50.6939 | 3.5 |
| 2930 | BNN07 | 2004† | 6.7129 | 51.0435 | 5.1 |
| HAMFR2010-11 | W01 | 2010 | 7.5197 | 48.4669 | 4.9 |
| HD721 | W02 | 2010 | 8.6621 | 49.4153 | 4.8 |
| H170522 | W04 | 2001 | 8.2731 | 49.9526 | 4.2 |
| 11663 | W03 | 1986 | 8.2911 | 49.7954 | 4.8 |
| Harxheim | W05 | 2014 | 8.2743 | 49.9039 | 4.2 |
| 405 | W06.1 | 2009 | 8.5961 | 50.4806 | 4.7 |
| *‘Priska’ | W06.2 | 2021 | 8.6622 | 50.4947 | 5.2 |
| HH 14_1 | W07 | 2014 | 8.7181 | 50.4924 | 5.3 |
| Carla WöDö1 | W08 | 2022† | 8.8565 | 50.4122 | 4.4 |
| Friedberg | W09 | 2020 | 8.7357 | 50.3198 | 3.6 |
| H170450 | W11 | 2014† | 8.5032 | 50.0816 | 4.7 |
| MKK49-1 | W12.1 | 2012 | 8.8203 | 50.1640 | 4.7 |
| MKK24987 | W12.2 | 2020 | 8.7948 | 50.1776 | 4.2 |
| 7222895 | W13.1 | 2020 | 8.3727 | 50.0194 | 5.5 |
| Hochheim 2016 | W13.2 | 2016 | 8.3727 | 50.0194 | 4.6 |
| H180052 Bayern | W14 | 2019 | 9.9956 | 49.8102 | 3.8 |
| H150228 | C1 | 2015 | 10.032 | 52.2050 | 4 |
| Göttingen | C2 | 2022 | 9.9530 | 51.5592 | 3.5 |
| H230684 | C3 | 2017 | 10.66 | 51.1352 | 4.6 |
| H230685 | C4.1 | 2017 | 11.0156 | 50.9910 | 4 |
| Hock Erfurt | C4.2 | 2020 | 11.0156 | 50.9910 | 4.8 |
| Etgersleben | C5 | 2022 | 11.4148 | 51.9758 | 5.2 |
| Prosigk | C6 | 2019 | 12.0407 | 51.6918 | 4.6 |
| H150139 | P1 | 2008 | 17.2840 | 49.5731 | 5.3 |
| H150052 | P2 | 2008 | 17.2840 | 49.5731 | 4.5 |
| H150059 | P3 | 2008 | 17.2840 | 49.5731 | 5 |
| H150196 | P4 | 2008 | 17.2840 | 49.5731 | 5.1 |


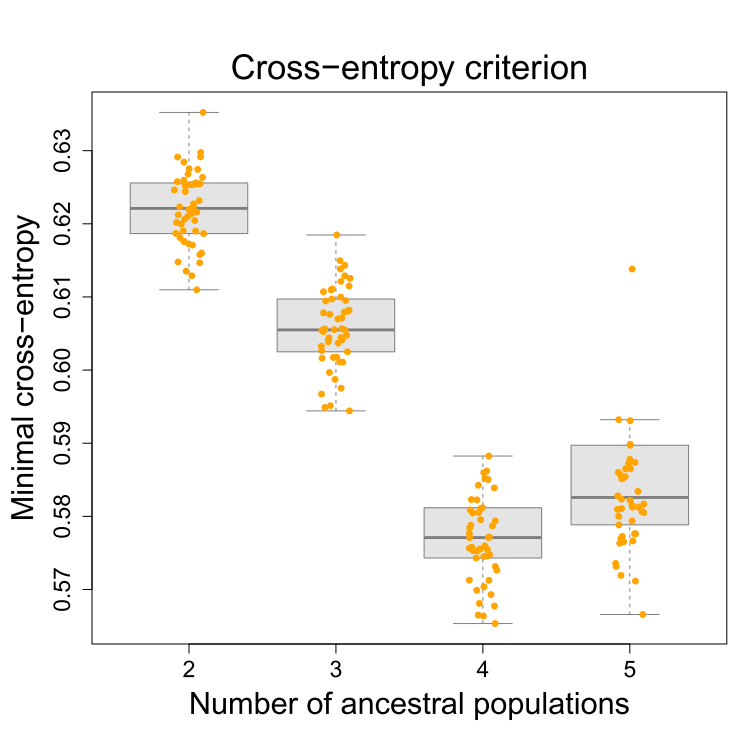


**Suppl. Fig. 2.** Results from a cross-entropy analysis. At K = 4 the entropy reaches a minimum.

Tab. S4**.** Comparison of de novo hamster genomes and reference species used as a reference by GeMoMa, common name, scientific name, and NCBI accessions.

| Species | Length | Contigs | Contig N50 | Reference |
| --- | --- | --- | --- | --- |
|  | Gbp | number | Mbp | Accession Nr. |
| **European hamster** | **2.9** | **686** | **267** | PRJNA1149967 |
| ***Cricetus cricetus*** |  |  |  |  |
| Chinese hamster | 2.40 | 204,464 | 0.039 | Lewis et al., 2013 |
| *Cricetulus griseus* |  |  |  | GCF_000223135.1 |
| Golden hamster | 2,46 | 7040 | 9.472 | Harris et al., 2022 |
| *Mesocricetus auratus* |  |  |  | GCF_017639785.1 |
| Desert hamster | 2.38 | 2079 | 25.78 | Andreotti et al., 2022 |
| *Phodopus roborovskii* |  |  |  | GCF_943737965.1 |
| Siberian hamster | 2.20 | 741 | 55.88 |  |
| *Phodopus sungorus* |  |  |  | GCA_030556225.1 |
| Mouse  *Mus musculus* |  |  |  | GCF_000001635.27 |
| Human  *Homo sapiens* |  |  |  | GCF_009914755.1 |

| **Tab. S5.** Annotation statistics   \| Annotation results \| statistic \| \| --- \| --- \| \| Number of gene \| 24507 \| \| Number of mrna \| 44946 \| \| Number of cds \| 44946 \| \| Number of exon \| 475728 \| \| Number of exon in cds \| 475728 \| \| Number of intron in cds \| 430782 \| \| Number of intron in exon \| 430782 \| \| Number gene overlapping \| 105 \| \| Number of single exon gene \| 2805 \| \| Number of single exon mrna \| 3060 \| \| mean mrnas per gene \| 1,8 \| \| mean cdss per mrna \| 1 \| \| mean exons per mrna \| 10,6 \| \| mean exons per cds \| 10,6 \| \| mean introns in cdss per mrna \| 9,6 \| \| mean introns in exons per mrna \| 9,6 \| \| Total gene length (bp) \| 439694237 \| \| Total mrna length (bp) \| 1053380486 \| \| Total cds length (bp) \| 77639562 \| \| Total exon length (bp) \| 77639562 \| \| Total intron length per cds (bp) \| 975740924 \| \| Total intron length per exon (bp) \| 975740924 \| \| mean gene length (bp) \| 17941 \| \| mean mrna length (bp) \| 23436 \| \| mean cds length (bp) \| 1727 \| \| mean exon length (bp) \| 163 \| \| mean cds piece length (bp) \| 163 \| \| mean intron in cds length (bp) \| 2265 \| \| mean intron in exon length (bp) \| 2265 \| \| Longest gene (bp) \| 381881 \| \| Longest mrna (bp) \| 381881 \| \| Longest cds (bp) \| 25920 \| \| Longest exon (bp) \| 22731 \| \| Longest cds piece (bp) \| 22731 \| \| Longest intron into cds part (bp) \| 59971 \| \| Longest intron into exon part (bp) \| 59971 \| \| Shortest gene (bp) \| 45 \| \| Shortest mrna (bp) \| 45 \| \| Shortest cds piece (bp) \| 3 \| \| Shortest intron into cds part (bp) \| 29 \| \| Shortest intron into exon part (bp) \| 29 \| |
| --- | --- | --- | --- | --- | --- | --- | --- | --- | --- | --- | --- | --- | --- | --- | --- | --- | --- | --- | --- | --- | --- | --- | --- | --- | --- | --- | --- | --- | --- | --- | --- | --- | --- | --- | --- | --- | --- | --- | --- | --- | --- | --- | --- | --- | --- | --- | --- | --- | --- | --- | --- | --- | --- | --- | --- | --- | --- | --- | --- | --- | --- | --- | --- | --- | --- | --- | --- | --- | --- | --- | --- | --- | --- | --- | --- | --- | --- | --- | --- | --- | --- | --- | --- | --- |

**Tab. S6.** Results generated from Repeatmasker

| Repeat Classes |
| --- |
| ============== |
| Total Sequences: 686 |
| Total Length: 2898698774 bp |
| Ancestral Repeats: 5680275 ( 1221972173 bp ) |
| Lineage Specific Repeats: 0 ( 0 bp ) |
| Class Count bpMasked %masked |
| ===== ===== ======== ======= |
| DNA 2399 324019 0.01% |
| Crypton 170 24176 0.00% |
| Crypton-A 94 12680 0.00% |
| Kolobok 156 22951 0.00% |
| MULE-MuDR 80 13374 0.00% |
| Merlin 114 14335 0.00% |
| PIF-Harbinger 187 30147 0.00% |
| PiggyBac 201 49108 0.00% |
| TcMar 107 18553 0.00% |
| TcMar-Mariner 813 180003 0.01% |
| TcMar-Pogo 18 2200 0.00% |
| TcMar-Tc1 153 24783 0.00% |
| TcMar-Tc2 1234 240995 0.01% |
| TcMar-Tigger 24650 5495665 0.19% |
| hAT 902 160074 0.01% |
| hAT-Ac 471 104832 0.00% |
| hAT-Blackjack 2842 401302 0.01% |
| hAT-Charlie 77691 14840914 0.51% |
| hAT-Tag1 305 62199 0.00% |
| hAT-Tip100 9024 1776145 0.06% |
| hAT-hAT19 10 956 0.00% |
| LINE -- -- -- |
| CR1 3382 612590 0.02% |
| Dong-R4 123 23333 0.00% |
| I-Jockey 18 3471 0.00% |
| L1 895248 490755807 16.93% |
| L1-Tx1 35 12792 0.00% |
| L2 16917 3041070 0.10% |
| RTE-BovB 278 50400 0.00% |
| RTE-X 1383 257964 0.01% |
| LTR 9141 1087283 0.04% |
| DIRS 25 1333 0.00% |
| ERV1 170613 60626178 2.09% |
| ERVK 751870 214599535 7.40% |
| ERVL 218064 38268617 1.32% |
| ERVL-MaLR 674397 121377498 4.19% |
| Gypsy 1426 239268 0.01% |
| PLE 39 7655 0.00% |
| RC -- -- -- |
| Helitron 272 50244 0.00% |
| Retroposon 245 40412 0.00% |
| L1-dep 29609 6756970 0.23% |
| sno 2548 564792 0.02% |
| sno-L1 14 1214 0.00% |
| SINE -- -- -- |
| 5S-Deu-L2 267 31084 0.00% |
| 5S-RTE 53 2499 0.00% |
| 7SL 35404 2368348 0.08% |
| Alu 671844 70783376 2.44% |
| B2 351746 52362866 1.81% |
| B4 502730 61253872 2.11% |
| ID 69888 4307102 0.15% |
| MIR 40512 4827749 0.17% |
| tRNA 4724 274002 0.01% |
| tRNA-Deu 49 7337 0.00% |
| tRNA-RTE 609 46865 0.00% |
| Unknown 93882 11052816 0.38% |
| --------------------------------- |
| total interspersed 4668976 1169495753 40.35% |
|  |
| Low_complexity 88256 4974959 0.17% |
| Satellite 38390 4074620 0.14% |
| Y-chromosome 1097 67388 0.00% |
| Simple_repeat 867412 41970327 1.45% |
| rRNA 1176 265139 0.01% |
| scRNA 6702 453297 0.02% |
| snRNA 3644 389929 0.01% |
| srpRNA 541 65452 0.00% |
| tRNA 4081 215309 0.01% |
| --------------------------------------------------------- |
| Total 5680275 1221972173 42.16% |

**Tab. S7.** Qualimap/MultiQC - Summary table of mapping and coverage metrics for all sequenced individuals.

| SampleID1 | Average GC content | Median insert size | Fraction of reads  Fraction of reads 1x | Fraction of reads  Fraction of reads 5x | Fraction of reads  Fraction of reads 10x | Median coverage | Mean coverage | General error rate | Percentage aligned | Mapped reads | Total reads |
| --- | --- | --- | --- | --- | --- | --- | --- | --- | --- | --- | --- |
| BNN01 | 41.2303273 | 306 | 99.4977786 | 93.5612315 | 59.7708602 | 11 | 13.1482 | 1.03 | 97.3435583 | 233.411815 | 239.78147 |
| BNN02 | 41.5648437 | 325 | 99.5721487 | 94.4431454 | 59.8014968 | 11 | 12.3376 | 1.02 | 96.9111237 | 219.456638 | 226.451443 |
| BNN03 | 41.4295012 | 312 | 99.6901171 | 98.3706034 | 86.1596129 | 15 | 17.1722 | 0.91 | 97.3763331 | 304.287602 | 312.486199 |
| BNN04 | 41.5890493 | 318 | 99.6369747 | 98.0350931 | 84.0634578 | 14 | 16.3406 | 0.84 | 97.3337886 | 286.933646 | 294.793463 |
| BNN05 | 42.2926408 | 315 | 99.5899609 | 94.6903285 | 63.0177014 | 11 | 13.5999 | 1.01 | 97.3367594 | 243.203729 | 249.85805 |
| BNN06.1 | 41.3468883 | 329 | 99.5613489 | 92.894916 | 51.4699387 | 10 | 11.4693 | 0.9 | 97.4376326 | 203.126583 | 208.468307 |
| BNN06.2 | 41.3466282 | 290 | 99.6472475 | 93.411897 | 54.6671456 | 10 | 12.2878 | 1.01 | 91.5502807 | 221.00167 | 241.399227 |
| BNN07 | 40.8130698 | 283 | 99.6286904 | 97.4782566 | 83.292207 | 15 | 18.3065 | 1.11 | 97.4422319 | 326.917662 | 335.498947 |
| C1 | 41.4078575 | 320 | 99.6115898 | 94.9463636 | 62.0041396 | 11 | 13.2301 | 1.02 | 97.3925399 | 234.00209 | 240.266955 |
| C2 | 41.4834712 | 322 | 99.3301649 | 92.4489034 | 50.4140825 | 10 | 11.9098 | 1.13 | 97.307644 | 212.477589 | 218.356524 |
| C3 | 41.5165009 | 300 | 99.7076838 | 97.0021832 | 74.3634864 | 12 | 15.3616 | 1.02 | 97.5132634 | 271.908871 | 278.842961 |
| C4,1 | 41.5001368 | 306 | 99.5804328 | 95.5735207 | 65.6502836 | 11 | 13.9869 | 1.01 | 96.9546401 | 247.046185 | 254.805943 |
| C4,2 | 41.6471625 | 294 | 99.7224243 | 97.6357064 | 79.2581722 | 13 | 16.4177 | 1.08 | 97.6342927 | 289.313865 | 296.324024 |
| C5 | 41.7003657 | 291 | 99.6766469 | 97.9747577 | 84.0032218 | 14 | 17.5308 | 1.02 | 97.6832081 | 308.463593 | 315.779548 |
| C6 | 41.5307962 | 318 | 99.6611111 | 97.2646915 | 77.3348009 | 13 | 15.7002 | 0.99 | 97.4840772 | 277.008695 | 284.157888 |
| P1 | 41.7449671 | 309 | 99.509772 | 97.3167072 | 83.2500301 | 14 | 16.674 | 1.04 | 97.4581792 | 294.476115 | 302.156389 |
| P2 | 41.5714703 | 302 | 99.4403434 | 96.3570925 | 73.4983637 | 12 | 14.8224 | 1.06 | 97.4999085 | 262.066852 | 268.786767 |
| P3 | 41.6411016 | 320 | 99.4699619 | 97.2284296 | 81.1514797 | 14 | 16.089 | 1.02 | 97.1931473 | 285.029478 | 293.260879 |
| P4 | 41.6450718 | 310 | 99.4901357 | 96.572699 | 80.1285603 | 14 | 15.6031 | 0.92 | 97.6376438 | 274.308512 | 280.945444 |
| Priska | 43.2265532 | 495 | 99.9827589 | 99.8237621 | 99.1654847 | 34 | 41.7356 | 0.75 | 96.8381996 | 894.673399 | 923.884792 |
| W01 | 40.9055655 | 290 | 99.6807041 | 97.3416728 | 78.8985612 | 13 | 16.8936 | 1.17 | 97.5295781 | 301.318754 | 308.951151 |
| W02 | 41.1038233 | 299 | 99.7415024 | 97.8422934 | 80.5068568 | 13 | 16.7227 | 1.01 | 97.600964 | 296.311592 | 303.594944 |
| W03 | 41.3883222 | 326 | 99.6767826 | 95.7795194 | 66.0262707 | 11 | 13.9559 | 1.05 | 97.1984284 | 247.995999 | 255.144042 |
| W04 | 41.5330145 | 298 | 99.805016 | 97.8664229 | 79.9880972 | 13 | 16.4079 | 1.08 | 96.8375852 | 290.596672 | 300.086657 |
| W05 | 41.4782057 | 326 | 99.6548249 | 96.2016657 | 67.8733203 | 11 | 14.0828 | 0.97 | 97.2938391 | 250.230808 | 257.190805 |
| W06.1 | 41.5160633 | 300 | 99.8401775 | 97.9019538 | 78.2438155 | 13 | 15.8039 | 0.88 | 97.601073 | 277.69086 | 284.516196 |
| W07 | 41.4052213 | 308 | 99.7506657 | 98.5246933 | 87.0557382 | 15 | 18.2795 | 0.96 | 97.4854173 | 324.63991 | 333.013818 |
| W08 | 41.5350771 | 307 | 99.8084835 | 97.3596504 | 73.3491895 | 12 | 15.0737 | 0.98 | 97.5326028 | 268.320262 | 275.108276 |
| W09 | 41.456175 | 318 | 99.6893851 | 93.9893663 | 54.5848187 | 10 | 12.3904 | 1.1 | 97.276863 | 221.703747 | 227.91005 |
| W11 | 41.4232417 | 308 | 99.7506916 | 97.742863 | 78.875608 | 13 | 16.0626 | 0.98 | 97.5720085 | 283.999519 | 291.066591 |
| W12.1 | 41.536194 | 306 | 99.757946 | 97.8363165 | 79.1549898 | 13 | 16.3715 | 0.95 | 97.2967547 | 289.208638 | 297.243869 |
| W12.2 | 41.2496348 | 312 | 99.7233635 | 96.9114746 | 71.2705608 | 12 | 14.5324 | 1.02 | 96.9392531 | 259.323814 | 267.511669 |
| W13.1 | 41.4774924 | 305 | 99.6814837 | 98.4724447 | 88.4837864 | 15 | 18.5884 | 0.92 | 97.5725362 | 327.239492 | 335.380738 |
| W13.2 | 41.4569028 | 302 | 99.7311189 | 97.3052952 | 75.4458819 | 12 | 15.243 | 0.92 | 97.5334406 | 268.021772 | 274.799874 |
| W14 | 40.8700354 | 308 | 99.7070903 | 94.6725404 | 61.2899605 | 11 | 13.5706 | 1.13 | 97.2212577 | 243.483048 | 250.442191 |
